# Factors affecting practices of recently delivered women on maternal and neonatal health care in selected rural areas of Bangladesh

**DOI:** 10.1186/s12884-023-05998-4

**Published:** 2023-09-27

**Authors:** Zerin Jannat, Md. Wazed Ali, Nurul Alam, Md. Jasim Uddin

**Affiliations:** Health Systems and Population Studies Division, icddr,b, 68- Shaheed Tajuddin Ahmed Sarani, Mohakhali, Dhaka, 1212 Bangladesh

**Keywords:** Recently delivered women, Rural Bangladesh, Maternal healthcare, Neonatal healthcare

## Abstract

Bangladesh has made laudable progress in maternal and child health (MCH). Maternal and child mortalities have reduced substantially accompanied by stellar rise in immunization and contraceptive prevalence rate (CPR). However, such success is distributed unevenly throughout and the country is among one of the top ten countries with highest number of neonatal and under-five children mortalities. Rural Bangladesh is home to more than half of the country’s total population. Yet, disparity in access to healthcare services and information are overt in these areas. Utilization of maternal health services (MHS) is low whereas maternal and child mortalities are high in the rural areas. Thus, this cluster randomized cross sectional study was conducted with the aim to observe the practices that rural women followed in regards to maternal and child health and factors that affected these practices. Primary data was collected from 550 respondents using a structured questionnaire within the time period September—October 2019. All our participants were recently delivered women (RDW), defined in our study as women of reproductive age (15–49 years) who had delivered a child recently, i.e. 12 months prior (September 2018 – August 2019) the data collection. We conducted logistic regression and multivariate analysis to analyze data. Results from this study depict that while 96.3% of RDW opted for ANC visits and 99.1% fed colostrum to their newborn, fewer have had institutional deliveries and the number of RDW who had PNC was only 64.7%. Education was found to be the most prominent factor that affected practices employed by RDW. The more educated a respondent was, the greater the chance was of her engaging in appropriate maternal and child health practices. The RDW preferred and visited private facilities the most to obtain healthcare services with private medical doctors being one of the prime sources of healthcare information for the respondents. On the contrary, monthly expenditure exerted no statistically significant impact on the aforementioned practices. Thus, results of our study imply that interventions enhancing education and health knowledge of women and engaging private sector be designed for improving maternal and neonatal health care in rural areas of Bangladesh.

## Introduction

Bangladesh emerged as a sovereign nation in 1971 [1]. The country’s economy was in tatters at its birth, branded as second poorest nation in the world [2]. Over the past five decades, however, Bangladesh made remarkable progress in poverty reduction and development. Formerly dubbed ‘basket case’, the country aspires to become an upper-middle income economy by 2031 [3]. Notably, another arena in which Bangladesh made commendable progress is the health sector [4].

Maternal deaths declined significantly; from 574 deaths per 100,000 live births in 1990 to 196 per 100, 000 live births in 2016 [5]. Such success has especially been enhanced by the stellar sevenfold rise in contraceptive prevalence rate (CPR); from 8% in 1975 to 62% in 2017 with the total fertility rate (TFR) decreasing from 6.8 to 2.3 within the same time frame [6, 7]. The infant mortality rate has also declined significantly from 64.2 deaths per 1,000 live births in 1990 to 20.1 per 1,000 in 2018 [8]. Under-five mortality reduced from 143.8 per 1,000 livebirths in 1990 to 32.4 per 1,000 in 2017 [9]. These reductions have been enhanced by the exemplary implementation of Expanded Program on Immunization (EPI). Dubbed as an exceptional health performer, Bangladesh was recognized in 2010 by the United Nations (UN) for commendable progress towards Millennium Development Goal (MDG) 4 in child mortality and for being on-track to achieve the maternal mortality reduction goals of MDG 5 [10]. However, such laudable improvements in health are not spread evenly throughout the country.

Maternal mortality ratio (MMR) continues to be unacceptably high and lives of approximately 14 mothers are lost every day from complications related to pregnancy and childbirth in Bangladesh [11, 12]. Furthermore, the country belongs to the list of top 10 countries with highest number of neonatal and under-five children mortalities [12]. The CPR and total fertility rate have stalled and one-third of the pregnancies is unintended, ensuing eventuate from unmet needs for family planning (FP), FP method discontinuation and switching of the methods [13, 7].

The rural Bangladesh is abode to approximately 61% of the total population [14]. Data from the Bangladesh Demographic Health Survey (BDHS) 2017–18 depict that 71% of the rural women face difficulty in getting access to health care compared to 58% of their urban counterparts [6]. Utilization of maternal health services such as antenatal care (ANC), institutional delivery, postnatal care (PNC) and utilization of FP services are alarmingly low despite the increasing availability and accessibility of modern health services in the rural areas [15]. Furthermore, both the MMR and child mortality have been reported to be higher in rural areas compared to the urban areas [16].

Maternal health is inextricably linked to newborn health [17]. The healthcare decisions of mothers have substantial impact on child morbidity and mortality [18]. Thus, practices that mothers follow based on the healthcare information and affordability they possess play a vital role in maternal and newborn health. Furthermore, factors that affect these practices are momentous in developing health interventions to address MMR and child mortality, especially in rural settings. However, more need to be known about the practices that rural women engage in regards to maternal and neonatal health. Given such scenario, we conducted this study with rural recently delivered women (RDW), defined in our study as women of reproductive age (15–49 years) who had delivered a child recently, i.e. a year prior to data collection. Our study areas are located in Rangpur, one of the eight divisions that has highest number of neonatal and under 5 mortalities and reported greater incidents of early childbearing within the country [16, 19]. Our aim was to explore the practices of recently delivered women on maternal and neonatal health as well as factors that affect these practices.

## Methods

### Study population, study area and data

This was a cluster randomized cross sectional study. Based on primary data collected in 2019, the study population included ever-married women of reproductive age (15–49 years) who had recently given birth (elaborated below). We selected two rural areas for our study from Rangpur division of Bangladesh. Administratively, the whole country is divided into 8 divisions. These divisions branch out to districts and sub-districts. The sub-districts then further divide into unions (rural areas), municipalities (towns) and city corporations (metropolis). Given this scenario, our study areas were Nafanagar and Ishania unions from Bochaganj sub-district of Dinajpur. Notably, Dinajpur district was selected purposively since it is located in Rangpur division where deaths of neonatal and under5 children have been reported to be the highest in the country [16].

### Data

We collected data from September—October 2019. Data were collected by conducting face-to-face interviews with recently delivered women (RDW) of age group 15–49 years who delivered child in recent past, i.e. during 12 months (September 2018 – August 2019) prior to the interview. A pre-tested survey questionnaire was used to collect primary data on practices that these mothers followed in regards to maternal and child health (MCH) care and the factors affecting these practices. Notably, the survey questionnaire was pilot-tested prior to data collection to ensure its feasibility and applicability in rural Bangladesh setting. We employed eight experienced Field Research Assistants (FRAs), two field supervisors and one field coordinator for the data collection. Two teams were formed where each team comprised of one field supervisor and four FRAs. The experienced FRAs conducted in-person interview after completion of their intensive training. They collected data using electronic devices such as tablets equipped with application “KoBoToolbox”. Each interview took around 45 min. Data quality was checked through random uninformed spot-checks and 5% re-interview by supervisors and checking consistency across variables during data cleaning. To elaborate, the interviews conducted were checked in several steps to ensure data quality. Foremostly, data collectors themselves conducted cross-checking of the interviews they completed. The field supervisors then checked data quality via random uninformed spot-checks and conducted 5% re-interview. The field coordinator was responsible for maintaining the overall data quality control throughout the data collection period. All these steps were taken to minimize errors and potential source of bias including recall bias. Furthermore, we were scrupulous in avoiding social desirability or recall bias during data collection. Stated before, data were collected by experienced FRAs of icddr,b. Comprehensive training was provided to the interviewers which also included special focus on recall bias. The FRAs were trained how to obtain correct information from the respondents asking some questions, such as noticeable events, dates, history, etc. to minimize the recall bias.

#### Sampling and sample size

We estimated the minimum required sample size for selected health indicators. This was done in order to obtain a specified precision, i.e. a confidence interval of a specified width, around a single point estimate of ANC 4 + visits, facility delivery, and PNC within 48 h (Table 1). The formula used for the calculation of the sample size is:

**Table 1:** Minimum required sample size per stratum

**Name of health indicators**	**Prior estimates** **BDHS 2016–2018**	**Nafanagar & Ishania Unions**	**Remarks**
Pregnancy care (ANC 4 +)	47%	535	Required sample size is 550
Post-natal care (PNC) within 48 h	52%	536	
Delivery in health facilities in last birth	50%	537	

$$n=\;\frac{Z_a^2\;P\left(1-P\right)}{d^2}\;\times\;\mathrm{design}\;\mathrm{effect}\;\times\;\;\mathrm{factor}\;\mathrm{to}\;\mathrm{adjust}\;\mathrm{for}\;\mathrm{non}-\mathrm{response}\;\mathrm{rate}$$

where P = coverage (%) of selected health indicators according to preliminary results of the BDHS 2017–2018,

Z_α_ = 1.96 at α = 0.05 and d = half of the confidence interval, the design effect = 1.27 (the BDHS 2014 estimate of the design effect is 1.27 for mothers received medical assistance during delivery) and factor to adjust for a non-response rate of 10%.

The minimum required sample size per stratum to obtain ± 5% precision of the indicators, adjusting for the design effect of 1.27 and non-response rate of 10% mentioned in the table below:

The required sample size for estimating MCH indicators (ANC 4 + , facility deliver and PNC) was 550. The average population of a Union is 25,000–30,000. Given the crude birth rate of 20 per 1000 population per year (BDHS 2017–2018), there are 1,000–1,200 mothers in Nafanagar and Ishania Unions who have infants aged 0–11 months and are eligible to provide information on ANC, health facility delivery and PNC.

A Union is divided into 24 EPI outreach centres, each home to approximately 1,000–1,200 residents. The catchment area of the EPI outreach centre is well defined, known to the community and treated as ‘cluster’. A large number (n = 48) of EPI clusters in the two Unions enabled to follow a two-stage random cluster sampling for selection of the survey households with eligible respondents. The first stage was a random selection of EPI outreach centres from the list of outreach centres of the Unions, and the second stage was selection of households with eligible survey participants.

From the list of 48 EPI centres in Nafanagar and Ishania Unions, 18 clusters per Union (18*2 =   36) were randomly selected for the survey of the recently delivered women. Primarily, it was supposed that there would be around 15–16 live births born in a cluster in one year preceding the survey. All the respondents who had live births in a cluster during one year preceding the survey were interviewed.

#### Data analysis

We processed data to standardize the survey results. Data processing was conducted as responses were recoded according to the analytical approach. Depending on the question/ response scale design, we evaluated response of neutral response or evaluated as “no comment” response. We also recoded binary yes/no response to questions.

All the continuous variables were summarized by the minimum and maximum values, the arithmetic mean, standard deviation, median (interquartile range) depending on the distribution. All categorical variables were summarized by frequencies and percentages. For scoring knowledge, all the knowledge related questions of each type of information were considered, and multiple responses were converted into dummy variables and provided score 1 for each “yes” response and 0 for other responses, such as ‘no’, ‘don’t know’ and ‘not applicable’. Total score then was calculated by combining respective dummy variables. The mean score and standard deviation were calculated as well.

To investigate the influence of RDW background characteristics on their MNCH related practices binary logistic regression analysis was performed and reported as an odds ratio (OR) with 95% CI for both adjusted and unadjusted models. Crude/unadjusted odds ratios were obtained by considering the effect of only one independent variable. Whereas adjusted odds ratios were obtained by including all influential variables in the model. Results were interpreted as statistically significant at a p-value of < 0.05. The list of independent variables included age, level of education, monthly expenditure, study areas, and corresponding knowledge score. On the other hand, the dependent variables were practice on pregnancy care, delivery, PNC and newborn care. All statistical analyses were performed using the statistical package STATA (version 15).

## Results

In this section, we present the results of our analyses in tabular forms. For comprehension purposes, please note that public health facilities are defined as compilation of government Satellite Clinics, Community Clinics, Union Health and Family Welfare Center (UH&FWC), Upazila Health Complex (UHC), district hospital/sador hospital, Maternal and Child Welfare Center (MCWC), medical college hospital. Private health facilities are defined as compilation of private clinics/hospitals, doctors’ chamber. The NGO health facilities are defined as compilation of NGO satellite clinics, NGO clinics/hospital, and Mission hospital.

### Socio-demographic characteristics of RDW

We conducted interview with 287 and 276 women from Ishania and Nafanagar unions, respectively. The RDW in Ishania and Nafanagar were mostly young adults aged between 20–24 years and have had some secondary education as observed from Table 2. We found that the RDW have had some education. One-third of the respondents from both unions had completed secondary or higher level of education. Notably, only 4.2% and 3.3% of the women had no institutional education in Ishania and Nafanagar, respectively. The percentage of RDW who were housewives exceeded 90% in both the unions.

**Table 2:** Background characteristics of the RDW

	**Ishania** ***n* = 287**	**Nafanagar** ***n* = 276**	**Total** ***n* = 563**
**Age in year**			
≤ 19	62 (21.6)^a^	58(21)	120(21.3)
20–24	77 (26.8)	101(36.6)	178(31.6)
25–29	74 (25.8)	67(24.3)	141(25.0)
30–34	54 (18.8)	35(12.7)	89(15.8)
≥ 35	20 (7.0)	15(5.4)	35(6.2)
**Level of education**			
No education	12(4.2)	9(3.3)	21(3.7)
Primary incomplete	40(13.9)	31(11.2)	71(12.6)
Primary complete	32(11.1)	26(9.4)	58(10.3)
Secondary incomplete	113(39.4)	121(43.8)	234(41.6)
Secondary or above	90(31.4)	89(32.2)	179(31.8)
**Marital status**			
Currently married	284(99.0)	276(100.0)	560(99.5)
Separated/Abandoned/Widowed	3(1.0)	-	3(0.5)
**Occupation**			
House wife	272(94.8)	262(94.9)	534(94.8)
Govt. service	3(1.0)	2(0.7)	5(0.9)
Private service	5(1.7)	4(1.4)	9(1.6)
Business	4(1.4)	5(1.8)	9(1.6)
Student	3(1.0)	3(1.1)	6(1.1)
**Monthly income (BDT)**			
Less than 10,000	51 (17.8)	80 (29.0)	131(23.3)
10,000–20,000	197(68.6)	150(54.3)	347(61.6)
More than 20,000	39(13.6)	46(16.7)	85(15.1)
**Monthly expenditure (BDT)**			
Less than 10,000	146(50.9)	155(56.2)	301(53.5)
10,000–20,000	134(46.7)	115(41.7)	249(44.2)
More than 20,000	7(2.4)	6(2.2)	13(2.3)
**Number of living children**			
1	134(46.7)	119(43.1)	253(44.9)
2	108(37.6)	118(42.8)	226(40.1)
3	40(13.9)	31(11.2)	71(12.6)
4 or more	5(1.7)	8(2.9)	13(2.3)

^a^All values are presented as N (%)

The income of most RDW, 68.6% in Ishania and 54.3% in Nafanagar, ranged from Bangladeshi taka (BDT) 10,000–20,000 per month (i.e. ∼ USD 100). The mean expenditure per month for the interviewed RDW was less than BDT 10,000 (i.e. ∼ USD 100–200) for both unions. More than 40% the RDW had at least one child but the percentage of women with two children was higher in Nafanagar (42.8%) compared to Ishania (37.6%). Spouses of most RDW were day labourers and approximately one-third of the husbands of all RDW had completed secondary or higher level of education.

#### Healthcare decision making

The interviewed RDW possessed limited decision-making power in regards to health care and other affairs (data not shown). The number of respondents who did not have the power to decide their own health care needs exceeded 50% at both study sites. Only 10% of the RDW could independently decide and seek health care. Their ability to make household decisions unrelated to health was also low; 13–17%. Notably, while RDW from both the unions had low decision-making power, the scenario appears to be worse in Nafanagar compared to Ishania.

## Practices of RDW on maternal and neonatal health

### Maternal healthcare

#### Practice of RDW on ANC and danger signs of pregnancy

Table 3 shows that 96% of the RDW at both Unions had received ANC during their last pregnancy. In contrast, the percentage of respondents who had made the World Health Organization (WHO) recommended ANC 4 + visits during their last pregnancy was substantially low- 44.6% and 42.4%, in Ishania and Nafanagar, respectively. Notably, RDW at both the selected study sites tended to visit private healthcare facilities for ANC visits. Nearly nine out of ten women in Ishania and eight in ten women at Nafanagar went to private healthcare facilities for ANC during their last pregnancy. Advice related to pregnancy care, measuring blood pressure, and abdominal examination were main services that the RDW received during their last pregnancy.

**Table 3:** ANC visits by RDW and facilities visited for ANC during last pregnancy

	**Ishania** ***n* = 287**	**Nafanagar** ***n* = 276**	**Total** ***n* = 563**
**Received ANC at last pregnancy**			
Yes	277(96.5)^a^	265(96.0)	542(96.3)
No	10(3.5)	11(4)	21(3.7)
**Number of ANC visits**			
None	10(3.5)	11(4)	21(3.7)
1	40(13.9)	41(14.9)	81(14.4)
2	60(20.9)	40(14.5)	100(17.8)
3	36(12.5)	50(18.1)	86(15.3)
4 or more	128(44.6)	117(42.4)	245(43.5)
Can’t remember	13(4.5)	17(6.2)	30(5.3)
**Place of ANC checkup**^**b**^			
Public health facilities	106(38.3)	94(35.5)	200(36.9)
Private health facilities	243(87.7)	211(79.6)	454(83.8)
NGO health facilities	40(14.4)	31(11.7)	71(13.1)
Other (At home)	2(0.7)	6(2.3)	8(1.5)
**Type of ANC services received**^**b**^			
Pregnancy and labor related advices	263(95.0)	251(94.7)	514(94.8)
Measured blood pressure	269(97.1)	241(90.9)	510(94.1)
Abdominal examination	264(95.3)	244(92.1)	508(93.7)
Gave iron tablet/medicine	258(93.1)	246(92.8)	504(93.0)
Ultrasonogram	257(92.8)	232(87.6)	489(90.2)
Measured weight/Height	242(87.4)	234(88.3)	476(87.8)
Urine tested	239(86.3)	216(81.5)	455(84.0)
Blood tested	239(86.3)	211(79.6)	450(83.0)
TT vaccination	151(54.5)	142(53.6)	293(54.1)

^a^All values are presented as N (%)

^b^Multiple responses

Table 4 shows results obtained from logistic regression analysis. According to the results, the RDW of age group 30–34 years and ≥ 35 years, (aOR = 2.4, 95%CI: 1.2, 4.8) and (aOR = 4.3,95%CI: 1.5, 12.5), were more likely to conduct ANC4 + visits compared to RDW with age group 20–24 years. The RDW who did not complete primary and secondary education were respectively 70% (aOR = 0.3,95%CI: 0.2, 0.7) and 50% (aOR = 0.5,95%CI: 0.3, 0.9) less likely to have ANC 4 + visits compared to those who had completed secondary education or above. It can, thus, be opined that the respondents with low level of education were less likely were make ANC 4 + visits.

**Table 4:** Logistic regression analysis of receiving ANC4 + visits during last pregnancy with background characteristics of RDW and their knowledge score

**Characteristics**	**ANC4 + visits (*n* = 245)**			
	**n(%)**	**Crude odds ratio** **(95% CI)**	**Adjusted odds ratio** **(95% CI)**	***p*-value**
**Age in year**				
≤ 19	54(22.0)	1.1(0.7–1.8)	1.1(0.7–1.9)	0.652
20–24	74(30.2)	Reference (Ref)	Ref	
25–29	59(24.1)	1.0(0.6–1.6)	1.1(0.6–1.8)	0.787
30–34	42(17.1)	1.3(0.8–2.1)	2.4(1.2–4.8)	**0.009**
≥ 35	16(6.5)	1.2(0.6–2.5)	4.3(1.5–12.5)	**0.008**
**Level of education**				
No education	8(3.3)	0.4(0.2–1.0)	0.7(0.3–2)	0.534
Primary incomplete	17(6.9)	0.2(0.1–0.4)	0.3(0.2–0.7)	0.003
Primary complete	20(8.2)	0.3(0.2–0.6)	0.6(0.3–1.1)	0.098
Secondary incomplete	92(37.6)	0.4(0.3–0.6)	0.5(0.3–0.9)	0.009
Secondary or above	108(44.1)	Ref	Ref	
**Monthly expenditure (BDT)**				
Less than 10,000	122(49.8)	0.8(0.6–1.2)	1.2(0.7–1.8)	0.542
10,000–20,000	112(45.7)	Ref	Ref	
More than 20,000	11(4.5)	6.7(1.5–30.9)	8(0.9–71.2)	0.063
**Number of living children**				
1 and 2	221(90.2)	Ref	Ref	
3 or above	24(9.8)	0.5(0.3–0.8)	0.3(0.1–0.6)	0.001
**Study Areas**				
Ishania	128(47.8)	Ref	Ref	
Nafanagar	117(100)	0.9(0.7–1.3)	1.2(0.8–1.8)	0.360
**Knowledge score about ANC visits during pregnancy (range:0–4)**		2.1(1.5–3.0)	1.8(1.2–2.6)	0.005
**Knowledge score about type of information knew about ANC (range:2–10)**		1.3(1.2–1.5)	1.1(0.9–1.2)	0.352
**Knowledge score about type of information knew about danger signs of pregnancy (range:0–6)**		1.5(1.3–1.8)	1.4(1.1–1.7)	0.002

The knowledge scores of RDW, related to ANC visits during pregnancy, different information on danger signs of pregnancy were significantly associated with receiving four or more ANC visits. Our results showed that the odds of respondents making ANC4 + visits increased by a factor of 1.8 (aOR = 1.8,95%CI: 1.2, 2.6) from a unit rise in knowledge score related to ANC visits during pregnancy. Similarly, a unit increase in knowledge score, related to type of information known to respondents about danger signs of pregnancy, increased the odds of making ANC4 + visits by factor of 1.4 (aOR = 1.4,95%CI: 1.1, 1.7). We also found significant association of ANC4 + visits with the number of living children. RDW who had three or more children were 70% (aOR = 0.3,95%CI: 0.1, 0.6) less likely to have ANC4 + visits compared to those who had 1 or 2 children. We did not find any significant association of ANC4 + visits with monthly expenditure and study areas.

Notably, we also found that almost all women in Ishania Union and nine out of ten women in Nafanagar Union knew about ANC and danger signs of pregnancy from their family members, neighbors and friends. The medical doctors at private health centers were cited as source of information for more than one-third RDW in both the study Unions (data not shown).

#### Experience and practice of RDW on pregnancy related complications

Table 5 shows that 16% of all the RDW interviewed had faced complications during the last pregnancy. Results show that a greater number of women in Nafanagar (19.2%) had experienced complications compared to those in Ishania (12.9%) during their last pregnancy. Almost all women who experienced complications during last pregnancy sought treatment for the complications they faced. Notably, 97.3% and 96.2% of the RDW sought such treatment in Ishania and Nafanagar, respectively. The respondents preferred visiting private healthcare facilities foremost, followed by public facilities for their treatment.

**Table 5:** RDW experience of complications in last pregnancy and their status of delivery related complicacies and treatment received for the complications

	**Ishania** ***n* = 287**	**Nafanagar** ***n* = 276**	**Total** ***n* = 563**
**Complications in last pregnancy**			
Yes	37(12.9)^a^	53(19.2)	90(16.0)
No	250(87.1)	223(80.8)	473(84.0)
**Type of complications**^**b**^			
No complications	250(87.1)	223(80.8)	473(84.0)
Severe abdominal pain	15(5.2)	14(5.1)	29(5.2)
Severe Headache	2(0.7)	9(3.3)	11(2.0)
Severe Fever	3(1.1)	8(2.9)	11(2.0)
Extreme vomiting	4(1.4)	5(1.8)	9(1.6)
Bleeding	2(0.7)	6(2.2)	8(1.4)
No movement of baby	3(1.1)	5(1.8)	8(1.4)
Swelling/ edema	3(1.1)	4(1.5)	7(1.2)
Blurred Vision	-	6(2.2)	6(1.1)
Convulsion	1(0.4)	-	1(0.2)
**Received treatment for complications**			
Yes	36(97.3)	51(96.2)	87(96.7)
No	1(2.7)	2(3.8)	3(3.3)
**Place of receiving treatment**^**b**^			
Public health care facilities	5(13.9)	7(13.7)	12(13.8)
Private health care facilities	30(83.3)	44(86.3)	74(85.1)
NGO health care facilities	1(2.8)	-	1(1.2)
Other (village doctor, traditional practitioners)	3(8.3)	2(3.9)	5(5.8)
**Experienced complications during last delivery**			
Yes	28(9.8)^a^	24(8.7)	52(9.2)
No	259(90.2)	252(91.3)	511(90.8)
**Type of complications during last delivery**^**b**^			
Prolonged labor (> 20 h)	6(2.1)	5(1.8)	11(2.0)
Excessive bleeding	3(1.1)	4(1.5)	7(1.2)
Undilated cervix	3(1.1)	2(0.7)	5(0.9)
Rupture of membrane	3(1.1)	3(1.1)	6(1.1)
Severe headache/blurred vision	3(1.1)	1(0.4)	4(0.7)
Convulsions	2(0.7)	1(0.4)	3(0.5)
High Blood Pressure	1(0.4)	1(0.4)	2(0.4)
Placenta related complications	2(0.7)	2(0.7)	4(0.7)
High fever	2(0.7)	-	2(0.4)
Limb presentation during delivery	1(0.4)	1(0.4)	2(0.4)
Others	5(1.7)	3(1.1)	8(1.4)
**Visited doctor for the complications**			
Yes	23(82.1)	23(95.8)	46(88.5)
No	5(17.9)	1(4.2)	6(11.5)
**Facilities visited for treatment**^**b**^			
Public health facilities	4(17.4)	7(30.4)	11(23.9)
Private health facilities	19(82.6)	14(60.9)	36(70.6)
NGO health facilities	-	1(4.4)	1(2.2)
Other (Village doctor, traditional practitioners)	1(4.4)	2(8.7)	3(6.5)
**Cost of treatment of complications**			
Median (IQR)	5000 (1200–10000)	2500(1500–12000)	3500(1500–10000)
**Problems to get delivery treatment**			
Yes	1(4.3)	1(4.3)	2(4.3)
No	22(95.7)	22(95.7)	44(95.7)
**Type of problem**^**b**^			
Didn’t provide adequate service/ medicine	1(100.0)	-	1(50.0)
Service provider didn’t look after properly	-	1(100.0)	1(50.0)

^a^All values are presented as N (%)

^b^Multiple responses

One in ten women from the two sites reported having experiences of complications during their last delivery. About 82.1% women in Ishania and 95.8% in Nafanagar sought treatment from medical doctors for complications related to delivery. In terms of facilities visited, about eight in ten women in Ishania (82.6%) and six in ten women in Nafanagar (60.9%) visited private healthcare facilities to receive treatment for delivery related complications. Almost all the women in both Unions mentioned that they had no difficulty in availing treatment for delivery related complications. However, women in Ishania spent more money on treating delivery related complications than women in Nafanagar. The amounts spent were BDT5000 and BDT 2500, respectively, in the two Unions.

Table 6 shows the results of logistic regression analysis which depict a statistically significant association between RDW level of education and complications experienced during last delivery. Respondents with lower education level, i.e. secondary education incomplete or below, were more prone to experience complications during their previous childbirth. To elaborate, the RDW who had no education were 4.6 times (aOR = 4.6, 95% CI:1.1,19.5) more likely to experience complications during their last delivery compared to those who had secondary education or above. A similar scenario was observed for RDW who did not complete secondary level education RDW, they were 2.5 times (aOR = 2.5, 95% CI:1.1,6.0) more likely to experience complication during their previous delivery. We also found significant association between the knowledge score of RDW about preparations needed for safe delivery and the complications experienced during last pregnancy. One unit increase in knowledge decreased the odd of experiencing complication by factor 0.8 (aOR = 0.8, 95% CI:0.7,0.9). We did not find any significant association of complications experienced during last pregnancy with age in year, monthly expenditure, number of living children, study areas and knowledge score related to the type of danger signs during delivery.

**Table 6:** Logistic regression analysis of experiencing complications during last pregnancy by background characteristics and respective knowledge score

**Characteristics**	**Experienced complications during last delivery**			
	**n(%)**	**Crude odds ratio** **(95% CI)**	**Adjusted odds ratio** **(95% CI)**	***p*-value**
**Age in year**				
≤ 19	12(23.1)	1.4(0.6–3.2)	1.3(0.5–3.1)	0.594
20–24	13(25.0)	Reference (Ref)	Ref	
25–29	12(23.1)	1.2(0.5–2.7)	1(0.4–2.2)	0.909
30–34	9(17.3)	1.4(0.6–3.5)	0.8(0.3–2.3)	0.625
≥ 35	6(11.5)	2.6(0.9–7.5)	1.3(0.3–5.3)	0.670
**Level of education**				
No education	4(7.7)	5.0(1.4–18.4)	4.6(1.1–19.5)	0.040
Primary incomplete	8(15.4)	2.7(1.0–7.5)	2.0(0.7–6.5)	0.220
Primary complete	7(13.5)	2.9(1.0–8.5)	2.2(0.7–7.0)	0.190
Secondary incomplete	25(48.1)	2.6(1.1–5.8)	2.5(1.1–6.0)	0.036
Secondary or above	8(15.4)	Ref	Ref	
**Monthly expenditure (BDT)**				
Less than 10,000	22(42.3)	0.6(0.3–1.1)	0.6(0.3–1.3)	0.199
10,000–20,000	29(55.8)	Ref	Ref	
More than 20,000	1(1.9)	0.6(0.1–5.0)	1.0(0.1–9.4)	0.983
**Number of living children**				
1 and 2	37(71.2)	Ref	Ref	
3 or above	15(28.8)	2.6(1.4–4.9)	2.2(0.8–6)	0.109
**Study Areas**				
Ishania	28(53.8)	Ref	Ref	
Nafanagar	24(46.2)	0.9(0.5–1.6)	0.7(0.4–1.3)	0.287
**Knowledge score about type of danger signs during delivery (range:0–5)**		0.8(0.6–1.0)	1(0.7–1.4)	0.831
**Knowledge score about preparation needed for safe delivery (range:0–13)**		0.8(0.7–0.9)	0.8(0.7–0.9)	0.010

When questioned about their source of information on preparation for safe delivery more than 90% of all women in Ishania and Nafanagar reported that they received information on preparation for safe delivery from their family members, neighbors and friends. Medical doctors at private health facilities, on the other hand, were the source of information for about four in ten RDW (36.8% and 40.2%) in Ishania and Nafanagar Unions (data not shown).

#### Place of last delivery and person assisted during delivery

Table 7 presents the percentage distribution of women according to their place of last delivery and the person who conducted their last delivery. About 60% of the births were conducted at private clinics/hospitals whereas more than 20% child deliveries took place at home. Nearly 60% of births in Ishania and 50% in Nafanagar were attended by medical doctors at private facilities. The traditional birth attendants conducted more than 10% deliveries at home in both study areas.

**Table 7:** Place of last delivery and the person assisted the delivery

	**Ishania** ***n* = 287**	**Nafanagar** ***n* = 276**	**Total** ***n* = 563**
**Place of last delivery**			
At home	68(23.7)^a^	57(20.7)	125(22.2)
Union Health and Family Welfare Center (UH&FWC)	1(0.3)	-	1(0.2)
Upazila Health Complex (UHC)	33(11.5)	36(13)	69(12.3)
District sadar hospital	6(2.1)	2(0.7)	8(1.4)
Maternal and Child Welfare Center (MCWC)	5(1.7)	8(2.9)	13(2.3)
Medical college hospital	1(0.3)	2(0.7)	3(0.5)
Private clinic/hospital	170(59.2)	164(59.4)	334(59.3)
NGO clinic/hospital	2(0.7)	5(1.8)	7(1.2)
Others	1(0.3)	2(0.7)	3(0.5)
**Assistance during delivery**			
**Government**			
Family Welfare Assistant (FWA)	1(0.3)	-	1(0.2)
Family Welfare Visitors (FWV)	2(0.7)	6(2.2)	8(1.4)
Nurse/Midwife	35(12.2)	33(12)	68(12.1)
MBBS^b^ doctor	10(3.5)	9(3.3)	19(3.4)
**NGO**			
MBBS doctor	-	12(4.3)	12(2.1)
Paramedic/Nurse	1(0.3)	8(2.9)	9(1.6)
**Private**			
MBBS doctor	165(57.5)	139(50.4)	304(54)
Nurse	10(3.5)	12(4.3)	22(3.9)
**Others**			
Trained Traditional Birth Attendants (TTBA)	20(7)	15(5.4)	35(6.2)
Traditional Birth Attendants (TBA)	35(12.2)	40(14.5)	75(13.3)
Other (village doctor, family member)	8(2.8)	2(0.7)	10(1.8)

^a^All values are presented as N (%)

^b^Bachelor of Medicine and Bachelor of Surgery (MBBS)

#### Practice of RDW on PNC

Table 8 shows that more than 60% of the RDW received PNC after the birth of their last child in both study Unions. However, the number of women had PNC 4 + visits was substantially lower- 28.9% and 18.1% in Ishania and Nafanagar Unions, respectively. More than 90% of the women who did not receive PNC at both Unions, explained that they faced no problems/complications after childbirth and hence, did not need PNC. Private facilities were the most preferred and visited for PNC by the RDW. RDW in Ishania received greater amount of medicines compared to RDW in Nafanagar- 87% and 76%, respectively. Notably, the RDW received information on PNC foremost through family members, neighbors or friends, followed by private medical doctors (data not shown).

**Table 8:** Status of PNC among RDW

	**Ishania** ***n* = 287**	**Nafanagar** ***n* = 276**	**Total** ***n* = 563**
**Received PNC**			
Yes	191(66.6)^a^	173(62.7)	364(64.7)
No	96(33.4)	103(37.3)	199(35.3)
**Number of PNC visits**			
None	96(33.4)	103(37.3)	199(35.3)
1	36(12.5)	39(14.1)	75(13.3)
2	33(11.5)	34(12.3)	67(11.9)
3	37(12.9)	50(18.1)	87(15.5)
4 or more	83(28.9)	50(18.1)	133(23.6)
Can't remember	2(0.7)	-	2(0.4)
**Place of PNC visit**^**b**^			
Public health facilities	43(22.5)	33(19.1)	76(20.9)
Private health facilities	150(78.5)	141(81.5)	291(79.9)
NGO health facilities	1(0.5)	4(2.3)	5(1.4)
Other (Village doctor, traditional healers)	4(2.1)	4(2.3)	8(2.2)
**Type of PNC services**^**b**^			
Measured blood pressure	187(97.9)	162(93.6)	349(95.9)
Counseling for PNC	188(98.4)	157(90.7)	345(94.8)
Check the uterus	45(23.6)	67(38.7)	112(30.8)
Measured weight/height	36(18.8)	34(19.6)	70(19.2)
Medicine provided:			
Vitamin B-complex	166(86.9)	134(77.5)	300(82.4)
Iron tablet	165(86.4)	129(74.6)	294(80.8)
Antacid	125(65.4)	75(43.3)	200(54.9)
Medicine of itching	41(21.5)	10(5.8)	51(14)
Eye drop	2(1.1)	3(1.7)	5(1.4)
**Reasons for not having PNC**^**b**^			
No problem at all	92(95.8)	96(93.2)	188(94.5)
Cured spontaneously	27(28.1)	20(19.4)	47(23.6)
Less important	4(4.2)	1(0.9)	5(2.5)

^a^All values are presented as N (%)

^b^Multiple responses

Table 9 depicts results of logistic regression receiving PNC4 + visits at last pregnancy with RDW background characteristic and respective knowledge score. Interestingly, the RDW who had no institutional education and RDW who did not complete secondary level of education were 5.3 and 2.3 times more likely, respectively to make four or greater number of PNC visits compared to women who had secondary level of education or above. Receiving four times or more PNC visits were significantly associated with study areas and place of delivery. Women from Nafanagar were 50% (aOR = 0.5, 95% CI:0.3,0.8) less likely to receive PNC 4 + visits compared to women from Ishania. Regarding the place of delivery, the RDW who preferred government health facilities were 90% (aOR = 0.1, 95% CI: 0.1,0.3) less likely to receive PNC compared to those who used private healthcare facilities.

**Table 9:** Logistic regression of receiving PNC4 + visits at last pregnancy with RDW background characteristic and respective knowledge score

**Characteristics**	**PNC4 + (*n* = 133)**			
	**n(%)**	**Crude odds ratio** **(95% CI)**	**Adjusted odds ratio** **(95% CI)**	***p*-value**
**Age in year**				
≤ 19	25(18.8)	0.8(0.5–1.5)	0.7(0.4–1.4)	0.334
20–24	42(31.6)	Reference (Ref)	Ref	
25–29	37(27.8)	1.2(0.7–1.9)	1.2(0.6–2.2)	0.634
30–34	26(19.5)	1.3(0.7–2.4)	1.3(0.6–2.8)	0.489
≥ 35	3(2.3)	0.3(0.1–1.0)	0.4(0.1–1.5)	0.160
**Education level**				
No education	8(6.0)	2.2(0.8–5.7)	5.3(1.3–21.9)	0.020
Primary incomplete	10(7.5)	0.6(0.3–1.3)	1.5(0.6–4.0)	0.414
Primary complete	11(8.3)	0.8(0.4–1.8)	1.6(0.6–4.0)	0.327
Secondary incomplete	65(48.9)	1.4(0.9–2.2)	2.3(1.3–4.1)	0.005
Secondary or above	39(29.3)	Ref	Ref	
**Monthly expenditure (BDT)**				
Less than 10,000	71(53.4)	0.9(0.7–1.5)	1(0.5–1.7)	0.942
10,000–20,000	59(44.4)	Ref	Ref	
More than 20,000	3(2.3)	0.9(0.3–3.6)	0.6(0.1–2.5)	0.452
**Number of living children**				
1 and 2	118(88.7)	Ref	Ref	
3 or above	15(11.3)	0.7(0.4–1.2)	1(0.4–2.5)	0.980
**Study Areas**				
Ishania	83(62.4)	Ref	Ref	
Nafanagar	50(37.6)	0.5(0.4–0.8)	0.5(0.3–0.8)	0.008
**Place of delivery**				
Govt facilities	9(6.8)	0.2(0.1–0.4)	0.1(0.1–0.3)	< 0.001
Private facilities	124(93.2)	Ref	Ref	
**Knowledge score about PNC visits range = 0–4)**		1.8(1.5–2.2)	1.6(1.3–2.0)	< 0.001
**Knowledge score about type of information knew about PNC (range = 0–5)**		1.7(1.4–2.1)	1.5(1.1–2.0)	0.009
**Knowledge score about type of information knew about post-partum danger signs (range = 0–4)**		1.5(1.2–1.8)	0.9(0.7–1.2)	0.507

The RDW knowledge score related to PNC visits was associated with increased odds of their practice about four times or more PNC visits (aOR = 1.6, 95% CI: 1.3,2.0). Furthermore, knowledge related to particular information on PNC was significantly associated with increased odds of their practice about PNC visits (aOR = 1.5, 95% CI: 1.1,2.0). No significant association was found with age in year, monthly expenditure, number of living children, knowledge score about the type of information the women knew about post-partum danger signs.

## Neonatal healthcare

Our study also assessed the number of RDW who fed colostrum to their newborn and the results are shown in Table 10. This practice was found widespread among all the surveyed women in both areas. Only few women were unable to continue this practice due to insufficient supply of milk or other personal reasons (data not shown). In regards to newborn complications within 28 days of birth, about four in ten newborns in study Unions (41.5% and 44.2%, in Ishania and Nafanagar, respectively) had complications after their birth. Cold/cough was common among newborns followed by fever in both the Unions. Most RDW, approximately 90% and 80% in Ishania and Nafanagar, respectively went to health facilities for treatment of complications in the newborn. Nearly 60% mothers in both Unions went to private healthcare facilities for treating complications in the newborn. Notably, more than 90% of the RDW in both areas had heard about danger signs in newborn from their family members, neighbors and friends. Medical doctors at private facilities were also found to be a prominent source of information for approximately 34.8% and 38.0% women, respectively, in Ishania and Nafanagar Unions (data not shown).

**Table 10:** Practice on newborn care by RDW

	**Ishania** ***n* = 287**	**Nafanagar** ***n* = 276**	**Total** ***n* = 563**
**Feed colostrum to newborn**			
Yes	285(99.3)^a^	273(98.9)	558(99.1)
No	2(0.7)	3(1.1)	5(0.9)
**Complications of newborn**			
Yes	119(41.5)	122(44.2)	241(42.8)
No	168(58.5)	154(55.8)	322(57.2)
**Type of complications in 0–27 days**^**b**^			
Cold/cough	51(42.9)	43(35.3)	94(39.0)
Fever	35(29.4)	38(31.2)	73(30.3)
Skin rash	27(22.7)	25(20.5)	52(21.6)
Jaundice	16(13.5)	17(13.9)	33(13.7)
Flatulence	13(10.9)	15(12.3)	28(11.6)
Difficult or fast breathing	5(4.2)	7(5.7)	12(5.0)
Red or swollen eyes	3(2.5)	13(10.7)	16(6.6)
Pneumonia	8(6.7)	3(2.5)	11(4.6)
Poor sucking or feeding	4(3.4)	3(2.5)	7(2.9)
Did not pass stool	3(2.5)	3(2.5)	6(2.5)
Plus, bleeding, or discharge from the umbilical cord	2(1.7)	1(0.8)	3(1.2)
Continuous vomiting	4(3.4)	2(1.6)	6(2.5)
Diarrhea	2(1.7)	2(1.6)	4(1.7)
Convulsions/spasms/rigidity	1(0.8)	1(0.8)	2(0.8)
Lethargy/unconsciousness	1(0.8)	-	1(0.4)
Chest in drawing	1(0.8)	1(0.8)	2(0.8)
Baby did not cry	-	1(0.8)	1(0.4)
Difficult to wake up while sleep	1(0.8)	-	1(0.4)
Others	3(2.5)	8(6.6)	11(4.6)
**Had treatment for the complications**			
Yes	107(89.9)	99(81.1)	206(85.5)
No	12(10.1)	23(18.9)	35(14.5)
**Reasons for not seeking treatment**^**b**^			
Got cured spontaneously	11(91.7)	17(73.9)	28(80)
Brought medicine from pharmacy	-	4(17.4)	4(11.4)
Not given importance		1(4.4)	1(2.9)
Not affordable	2(16.6)	-	2(5.7)
**Place of receiving treatment**^**b**^			
Public health facilities	11(9.2)	22(18.0)	33(13.7)
Private health facilities	68(57.1)	71(58.2)	139(57.7)
Other (Village doctor, traditional practitioners)	37(31.1)	16(13.1)	53(22.0)
Did not receive treatment	12(10.1)	23(18.9)	35(14.5)

^a^All values are presented as N (%)

^b^Multiple responses

Findings of logistic regression analysis, shown in Table 11, show that RDW level of education was significantly associated with complications in newborn. Our results show that newborns born to RDW with higher level of education were less likely to experience complications. To elaborate, newborn of the RDW with a primary level of education were (aOR = 2.3, 95% CI: 1.2,4.4) more likely to experience complications compared to the newborn of RDW with secondary or higher level of education. However, we did not find any statistically significant association of complications in the newborn to be associated with RDW age, monthly expenditure, number of living children, study areas, knowledge score related to the type of information about newborn care and knowledge score related to the type of danger signs of newborn.

**Table 11:** Logistic regression analysis of complications of newborn with background characteristics of RDW and their knowledge score

**Characteristics**	**Complications of newborn (*n* = 241)**			
	**n(%)**	**Crude odds ratio** **(95% CI)**	**Adjusted odds ratio** **(95% CI)**	***p*-value**
**Age in year**				
≤ 19	46(19.1)	0.8(0.5–1.3)	0.9(0.6–1.5)	0.769
20–24	77(32)	Reference (Ref)	Ref	
25–29	62(25.7)	1.2(0.7–1.6)	1(0.7–1.7)	0.848
30–34	36(14.9)	0.9(0.5–1.5)	0.8(0.4–1.5)	0.505
≥ 35	20(8.3)	1.7(0.8–3.6)	1.4(0.6–3.3)	0.469
**Level of education**				
No education	11(4.6)	1.7(0.7–4.3)	1.4(0.5–3.8)	0.465
Primary incomplete	31(12.9)	1.2(0.7–2.1)	1.0(0.6–1.9)	0.898
Primary complete	36(14.9)	2.6(1.4–4.8)	2.3(1.2–4.4)	**0.011**
Secondary incomplete	94(39.0)	1.1(0.7–1.6)	1.0(0.7–1.6)	0.839
Secondary or above	69(28.6)	Ref	Ref	
**Monthly expenditure (BDT)**				
Less than 10,000	135(56)	1.2(0.8–1.7)	1.0(0.7–1.5)	0.990
10,000–20,000	99(41.1)	Ref	Ref	
More than 20,000	7(2.9)	1.8(0.6–5.4)	2.3(0.7–7.5)	0.183
**Number of living children**				
1and 2	197(81.7)	Ref	Ref	
3 and more	44(18.3)	1.6(0.9–2.5)	1.4(0.8–2.7)	0.254
**Study Areas**				
Ishania	119(49.4)	Ref	Ref	
Nafanagar	122(50.6)	1.1(0.8–1.6)	1.1(0.8–1.7)	0.490
**Knowledge score about type of information about new-born care (range = 1–7)**		0.9(0.8–1.1)	1.0(0.8–1.1)	0.642
**Knowledge score about type of danger signs of new-born (range = 0–6)**		1.0(0.9–1.2)	1.1(0.9–1.3)	0.288

## Discussion

In our study, we found female education to be of utmost importance for the RDW at the selected rural areas. Women with higher education were more likely to utilize MHS, a scenario consistent with different findings from different studies conducted in Bangladesh and Nepal [20–23]. Our results show that RDW with higher level of education were more likely to make ANC 4 + visits in contrast to their counterparts who have little to no education. This is similar to the findings of a study conducted in Nigeria which reported that educated women were two folds likely to make minimum number of recommended ANC visits compared to women with no education [24]. Our results also depict that RDW with higher education had experienced lesser complications during their last childbirth in contrast to their counterparts who have little to no education. Similarly, newborns of RDW with higher level of education were less likely to face complications. Given the findings, adopting policies that encourage and enhance education will be beneficial on many aspects. Greater education will help rural women to opt more for utilization of MHS and thus, led to significant improvement in maternal and neonatal health. Findings also showed that very few, i.e. less than 10%, respondents had the autonomy to make their own healthcare choices. Education empowers women and help rural women to engage in practices suitable for maternal and newborn health [25].

Possession of health knowledge exerted substantial impact on practices that the RDW employed in regards to utilization of MHS services. According to our findings from both areas, a unit increase in knowledge score related to ANC and danger signs of pregnancy significantly increased the probabilities of RDW making ANC 4 + visits. Similarly, the more knowledge the respondents had on preparations needed for safe delivery, the less they were to be at risk of facing pregnancy related complications. In regards to PNC, a unit increase in knowledge score of adequate PNC visits and PNC-specific information led to significant rise in the PNC 4 + visits made. Our findings illustrate that greater knowledge enables RDW to engage in suitable maternal and neonatal health practices. These results are also in par with the claims made in another study that health information enables expectant mothers to effectively utilize MHS, make apprised decisions in regards to their health, engage in appropriate health behaviors and enhance self-care [19].

Along with appropriate knowledge, place of delivery also affected the RDW on making PNC 4 + visits. Interestingly, about 90% of respondents who preferred government health facilities were less likely to return for a second PNC visit even though PNC is provided free of cost at these facilities. On the contrary, private health facilities were preferred most with private medical doctors being one of the prime sources of health information for the RDW. The percentage of RDW who opted for PNC 4 + visits was alarmingly low, i.e. 64.7% at our study areas. Thus, it is recommended that counseling be made available at public facilities with special focus on PNC for every mother that delivers a child there. It is known that a mother’s subsequent visits to a healthcare facility are prominently affected by the experience she has in terms of the care she receives [26]. In this regard, we further recommend that emphasis be given to privacy before and after childbirth, greater scope and ease of interaction with healthcare providers for mothers, address undocumented costs to encourage RDW in making PNC 4 + visits.

Monthly expenditure of our respondents did not exert any statistically significant impact over the choices and practices of the selected RDW. These results imply that financial costs may not always be the barrier that RDW face in engaging appropriate maternal and newborn childcare. There are other factors such as female education and empowerment, access to healthcare knowledge and quality of services received at the healthcare services play a vital role.

However, interventions focusing education and knowledge of women and engaging private sector may be designed for improving maternal and neonatal health care in rural areas of Bangladesh. This study had its limitation given that it was a very small-scale study conducted in only two unions of Bangladesh and thus, the findings cannot be generalized. Further large-scale research will enable to determine factors that exert the most significant impact and can be used to design appropriate interventions to improve maternal and neonatal healthcare practices in rural areas of Bangladesh.

## Data Availability

The datasets used and/or analysed during the current study are available from the corresponding author on reasonable request.
